# Identification and clinical significance of somatic oncogenic mutations in epithelial ovarian cancer

**DOI:** 10.1186/s13048-021-00876-z

**Published:** 2021-10-06

**Authors:** Takafumi Watanabe, Hideaki Nanamiya, Yuta Endo, Manabu Kojima, Shinji Nomura, Shigenori Furukawa, Shu Soeda, Hirosumi Tamura, Masae Ryufuku, Daisuke Tanaka, Takao Isogai, Jun-ichi Imai, Shinya Watanabe, Keiya Fujimori

**Affiliations:** 1 Department of Obstetrics and Gynecology, Fukushima Medical University, Fukushima, 960-1295 Japan; 2 Translational Research Center, Fukushima Medical University, Fukushima, 960-1295 Japan

**Keywords:** Epithelial ovarian cancer, Somatic oncogenic mutations, Molecular profiling, Prognostic biomarker

## Abstract

**Objective:**

Epithelial ovarian cancer (EOC) is a heterogeneous disease with diverse clinicopathological features and behaviors, and its heterogeneity may be concerned with the accumulation of multiple somatic oncogenic mutations. The major goals of this study are to systematically perform the comprehensive mutational profiling in EOC patients, and investigate the associations between somatic mutations and clinicopathological characteristics.

**Methods:**

A total of 80 surgical specimens were obtained from EOC patients who had previously undergone primary debulking surgery, and genomic DNAs were extracted from fresh-frozen tissues. We investigated mutational status in hot spot regions of 50 cancer-related genes by targeted next-generation sequencing using an Ion AmpliSeq Cancer Hotspot Panel v2 Kit.

**Results:**

Validated mutations were detected in 66 of the 80 tumors (82.5%). The five most frequently mutated genes were *TP53* (43.8%), *PIK3CA* (27.5%), *KRAS* (23.8%), *PTEN* (10%) and *CTNNB1* (10%). *PTEN* and *CTNNB1* mutations were associated with younger age. *PIK3CA1, KRAS* and *CTNNB1* mutations were observed in early-stage, whereas *TP53* mutations were more common in advanced stage. Significant associations were observed between TP53 mutation and serous carcinoma, and between *KRAS* mutation and mucinous carcinoma. Both *PIK3CA* mutation and *CTNNB1* mutation were also significantly associated with endometrioid and clear cell carcinoma. The patients with *PIK3CA* and *KRAS* mutations were significantly associated with favorable progression free survival (PFS). In particular, *PIK3CA* mutations had more significant associations with favorable PFS than *PIK3CA* wild-type in the endometrioid subtype (*P* = 0.012). Patients with mutations only in *TP53* were significantly associated with worse PFS.

**Conclusion:**

EOCs were heterogeneous at the genomic level and harbored somatic oncogenic mutations. Our molecular profiling may have the potential for becoming a novel stratification within histological subtypes of EOC. Further studies are needed to define molecular classification for improved clinical outcomes and treatment of EOC patients in future.

**Supplementary Information:**

The online version contains supplementary material available at 10.1186/s13048-021-00876-z.

## Introduction

Epithelial ovarian cancer (EOC) is the seventh most common cancer in women and the eighth most common cause of death from cancer in women worldwide [1, 2]. Recently, the incidence and mortality rates of patients with EOC have significantly increased. Although many surgical techniques and combination of chemotherapies have been widely promoted in clinical practice, the 5-year survival rate of EOC patients is 47% [3]. Therefore, a better understanding of potential molecular mechanisms provides opportunities for early diagnosis and optimal management of EOC.

EOC is a heterogeneous disease comprised of multiple subtypes which exhibit diverse clinicopathologic features and behaviors. Recently, a dualistic model has been proposed to divide EOC into two broad categories called type I and type II [4]. Type I cancers include low-grade serous cancer, endometrioid cancer, mucinous cancer, and clear cell cancer, which harbor somatic mutations such as *BRAF*, *KRAS* and *PTEN*, often with microsatellite instability (MSI). They are characterized by an indolent behavior diagnosed in early-stage, and arise in a stepwise process from borderline neoplasms [5–7]. Type II cancers are clinically aggressive and comprise high-grade serous carcinoma (HG-SC), carcinosarcomas, and undifferentiated carcinomas, which are associated with mutations in *TP53* and *BRCA1/2* [4, 5]. Although the classification of type I and II tumors better reflects the molecular diversity of EOC, the molecular biological heterogeneity of type I and II tumors has yet to be revealed.

Elucidating the relationship between genomic alterations and pathological factors can improve the clinical management and therefore survival rate of EOC patients, since it is now apparent that histological subtypes based primarily on morphology and immunohistochemistry tests are important for assessment in EOC. Several studies have revealed the genetic heterogeneity of each histological subtype such as serous carcinoma (SC), endometrioid carcinoma (EC), clear cell carcinoma (CCC) and mucinous carcinoma (MC) [8–11]. However, there have been few reports focusing on a systematic mutational landscape including all histological subtypes. In the present study, we performed comprehensive mutational profiling of EOC tumors using a cancer panel, and investigated the clinical significance of somatic oncogenic mutations.

## Materials and methods

### Clinical samples

A collection of specimens was obtained from EOC patients who had undergone primary debulking surgery at Fukushima Medical University Hospital between August 2013 and December 2017. Histopathological, clinical and treatment data were obtained from the patients’ clinical, operative and pathological records. Taxane-platinum combination chemotherapy was given to all patients who were candidates for adjuvant treatment. The current study was a retrospective study, using genomic analyses, and was approved by the ethics committee of Fukushima Medical University (No. 1953). Written informed consent was obtained from all patients.

### Next-Generation Sequencing (NGS) for hotspot regions in 50 cancer-related genes

Genomic DNAs were extracted from fresh-frozen tissue samples using ISOGEN reagent (Nippongene, Tokyo, Japan), according to the manufacturer’s instructions. The quality and quantity of each DNA sample were assessed using NanoDrop One (ThermoFisher Scientific, Waltham, MA, USA).

The NGS for genomic DNAs from each sample was performed using the Ion Ampliseq Cancer Hotspot Panel v2, which covers approximately 2800 mutational hotspot regions from 50 cancer-related genes [12, 13]. In brief, 10 ng of genomic DNAs extracted from 80 frozen tumor samples were used to construct barcoded DNA libraries utilizing an Ion Ampliseq Library Kit 2.0 (Thermo Fisher Scientific). The obtained libraries were optimized using an Ion Library Equalizer Kit (Thermo Fisher Scientific), and then sequenced using an Ion Personal Genome Machine or Ion S5XL platform (Thermo Fisher Scientific). The sequencing reads were aligned to the reference genome build hg19, GRCh37, and converted into BAM files using Ion Torrent Suite software (Thermo Fisher Scientific). Sequence variants were then called using Ion Reporter 5.0 (Thermo Fisher Scientific), according to the manufacturer’s instructions. The mean read depth of coverage in DNA sequencing was over 1500-fold.

### Statistical analysis

The associations between somatic mutations and stage or histology were evaluated using the chi-squared test. Somatic mutation frequency in each of the four histological subtypes, SC, EC, CCC, and MC was analyzed using the Kruskal-Wallis test. Progression free survival (PFS) and overall survival (OS) were evaluated as clinical outcomes. The Kaplan-Meier analysis with log-rank test was used to compare survival distributions. In all analyses, statistical significance was defined as *P* <  0.05. Statistical analysis was conducted using SPSS software version 25 (SPSS, Inc., Chicago, IL, USA).

## Results

### Patients characteristics

A total of 80 EOC patients who had undergone primary debulking surgery were enrolled in this study, and their clinicopathological characteristics are shown in Table 1. The median age at diagnosis was 60 years (range, 36–89 years), and 72 (90%) patients with high risk of recurrence underwent taxane-platinum chemotherapy postoperatively. Among the 80 tumors, there were 32 (40.0%) SC, 21 (26.3%) CCC, 20 (25.0%) EC, and seven (8.8%) MC (Table 1). All SC tumors in this study were HG-SC tumors. After a median follow-up of 41 months (range, 1–71 months), 56 (70%) patients were alive without clinical evidence of tumor. Recurrence was detected during the follow-up period in 43 (53.8%) patients: 17 (21.3%) patients were alive with disease; 24 (30%) patients died due to tumor progression.

**Table 1:** Clinicopathological characteristics and mutational status of patients with epithelial ovarian cancer

Characteristic	No. of cases	(%)
Median age, years (range)	60 (36-89)	
Age		
< 60	39	(48.8)
≥ 60	41	(51.3)
Stage		
I	22	(27.5)
II	12	(15.0)
III	38	(47.5)
IV	7	(10.0)
Histology		
SC	32	(40.0)
CCC	21	(26.3)
EC	20	(25.0)
MC	7	(8.8)
Mutational status		
None	14	(17.5)
One	36	(45.0)
Two or more	30	(37.5)
Median PFS (months) (95% CI)	39.2 (32.5-45.9)	
Median OS (months) (96% CI)	55.1 (49.6-60.4)	
3-year PFS	36	(45.0)
3-year OS	59	(73.8)

*SC* Serous carcinoma, *EM* Endometrioid carcinoma, *CCC* Clear cell carcinoma, *MC* Mucinous carcinoma

### Mutation frequencies in EOC

**Fig. 1:**
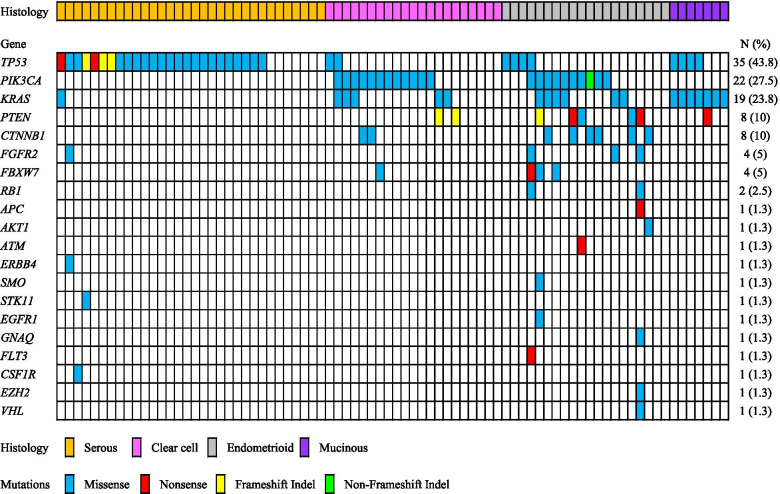
Summary of the associations between somatic mutations and histological features of epithelial ovarian cancer. All panels are placed on vertical tracks representing 80 individuals

We employed targeted NGS technology to explore somatic mutations occurring in EOC using 80 fresh-frozen tumors. A summary of the relationships between somatic mutations and histological characteristics is described in Fig. 1. Validated mutations were found in 66 of the 80 tumors (82.5%), and 30 of the 80 tumors (37.5%) harbored concurrent mutations in two or more genes (Table 1). Mutations were found in 20 among the 50 tumor-related genes in EOC tumors. Mutations were most frequently detected in *TP53* (43.8%), *PIK3CA* (27.5%) and *KRAS* (23.8%) (Fig. 1). *PTEN* (10%), *CTNNB1* (10%), *FBFR2* (5%) and *FBXW7* (5%) mutations were relatively frequent (Fig. 1 and Additional file 1). *RB1* (2.5%), *APC*, *AKT1*, *ATM*, *ERBB4*, *SMO*, *STK11*, *EGFR1*, *GNAQ*, *FLT3, CSF1R, EZH2* and *VHL* (1.3%) were minor mutations (Fig. 1 and Additional file 1). A total of 114 (mean, 1.43) mutations were detected; 98 (86%) missense mutations, 9 (7.9%) nonsense mutations, 6 (5.3%) frameshift indels and one (0.8%) non-frameshift indels (Fig. 1 and Additional file 1).

**Fig. 2:**
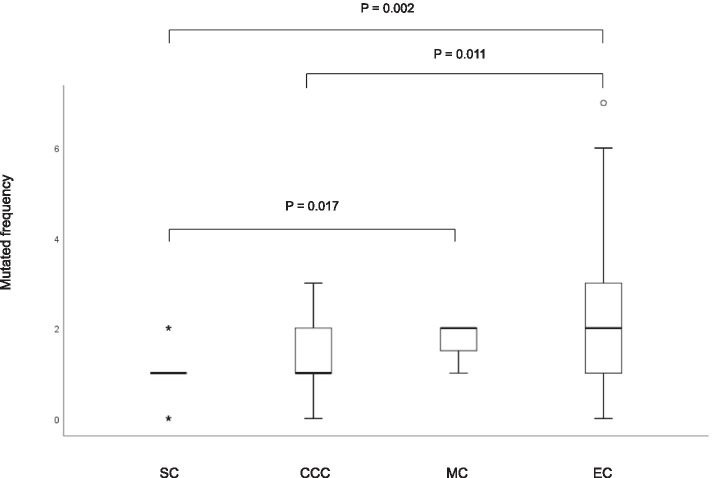
Box plot showing differences in mutation frequencies among four histological subtypes: SC (serous carcinoma), CCC (clear cell carcinoma), MC (mucinous carcinoma), EC (endometrioid carcinoma)

The mean (SD) of mutation frequency was 1.43 (1.26) in all EOC patients, 0.94 (0.61) in SC, 1.14 (0.83) in CCC, 1.71 (0.45) in MC and 2.4 (1.88) in EC. The association between mutation frequency and each histological subtype was investigated by the Kruskal-Wallis test. Significant difference was identified in mutation frequency between SC vs MC (*P* = 0.017), SC vs EC (*P* = 0.002) and CCC vs EC (*P* = 0.011) (Fig. 2).

### Associations of mutation status of *TP53*, *PIK3CA*, *KRAS*, *PTEN* or *CTNNB* with clinicopathological characteristics

A summary of the associations between each of the five most frequently mutated genes (*TP53*, *PIK3CA*, *KRAS, PTEN* and *CTNNB1*) and clinicopathological characteristics of the EOC patients is described in Table 2. Patients with *PTEN* and *CTNNB1* mutations had a significantly younger age (< 60) than those without these mutations (*P* = 0.02 and *P* = 0.021, respectively). *CTNNB1, PIK3CA* and *KRAS* mutations were significantly detected in early-stage EOC (*P* = 0.001, *P* = 0.004 and *P* = 0.009, respectively), whereas *TP53* mutations were significantly more common in advanced-stage EOC (*P* = 0.026). Significant associations between each of the five mutated genes (*TP53*, *PIK3CA*, *KRAS, PTEN* and *CTNNB1*) and histological subtypes were found as shown in Table 2.

**Table 2:** Frequency of *TP53*, *PIK3CA*, *KRAS*, *PTEN* and *CTNNB* mutations according to demographic and clinicopathological characteristics

	Age		*P* value	Stage		*P* value	Histology				*P* value
	< 60	≥ 60		I/II	III/IV		Serous	Clear cell	Endometrioid	Mucinous	
*TP53*											
WT	25	20		24	21		7	19	16	3	
MT	14	21	0.17	10	25	0.026	25	2	4	4	< 0.001
*PIK3CA*											
WT	26	32		19	39		32	9	10	7	
MT	13	9	0.25	15	7	0.004	0	12	10	0	< 0.001
*KRAS*											
WT	30	31		21	40		31	16	14	0	
MT	9	10	0.89	13	6	0.009	1	5	6	7	< 0.001
*PTEN*											
WT	31	41		31	41		32	19	15	6	
MT	8	0	0.002	4	4	0.71	0	2	5	1	0.033
*CTNNB1*											
WT	32	40		27	45		32	19	14	7	
MT	7	1	0.021	8	0	0.001	0	2	6	0	0.004

*WT* Wild-type, *MT* Mutant

### Prognostic role of clinicopathological factors and genomic mutations in EOC

**Fig. 3:**
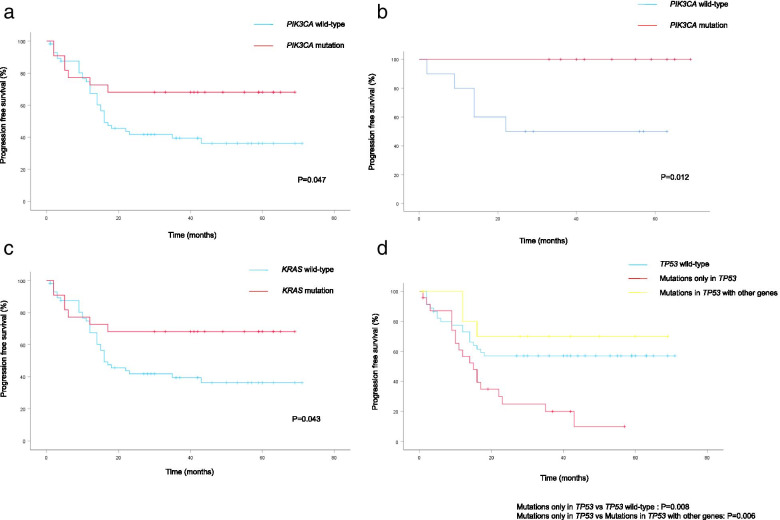
The Kaplan-Meier curves of progression free survival in patients with epithelial ovarian cancer. **a** *PIK3CA* mutations and wild-type. **b** *PIK3CA* mutations and wild-type in the patients with endometrioid carcinoma. **c** *KRAS* mutations and wild-type. **d** Mutations only in *TP53*, *TP53* wild-type, and mutations in *TP53* with other genes

The association between clinicopathological factors and survival is summarized in Table 3. The 3-year PFS and OS of the 80 patients were 50.0 and 75.0%, respectively. Late-stage, histological subtype, *PIK3CA* wild-type (WT) (Fig. 3a) and *KRAS* WT (Fig. 3c) were significantly associated with worse PFS (*P* <  0.001, *P* <  0.001, *P* = 0.047 and *P* = 0.043, respectively), while age, *TP53*, *PTEN* and *CTNNB1* were not. In particular, *PIK3CA* mutations had more significant associations with favorable PFS than *PIK3CA* WT in the EC subtype (*P* = 0.012) (Fig. 3b). Although *TP53* mutations did not influence the EOC survival, in the subgroup analysis, patients with mutations only in *TP53* were significantly associated with worse PFS compared to those with *TP53* WT (*P* = 0.008) or those with *TP53* mutation with other mutations (*P* = 0.006) (Fig. 3d) Regarding OS, as shown in Table 3, a significant association was only found with stage (*P* <  0.001), and age, histology, and mutations of *TP53*, *PIK3CA*, *KRAS*, *PTEN* and *CTNNB1* had no significant associations.

**Table 3:** The relationship of patients’ clinicopathological characteristics and somatic mutations with progression free survival and overall survival

	N	3-Year RFS (%)	*P*-value	3-Year OS (%)	*P*-value
Age (years)			0.17		0.31
< 60	38	55.3		78.9	
≥ 60	42	45.2		71.4	
Stage			< 0.001		< 0.001
I/II	34	79.4		93.9	
III/IV	46	28.3		63.0	
Histology			< 0.001		0.072
SC	32	17.9		67.9	
CCC	21	47.6		61.9	
EC	20	80		85.0	
MC	7	87.5		100	
*TP53*			0.088		0.74
WT	45	57.8		75.6	
MT	35	31.4		74.3	
*PIK3CA*			0.047		0.74
WT	58	43.1		75.9	
MT	22	68.2		72.7	
*KRAS*			0.043		0.11
WT	61	44.2		72.1	
MT	19	68.4		84.2	
*PTEN*			0.58		0.84
WT	72	48.6		75.0	
MT	8	62.5		75.0	
*CTNNB1*			0.59		0.89
WT	72	48.6		75.0	
MT	8	62.5		75.0	

*SC* Serous carcinoma, *EM* Endometrioid carcinoma, *CCC* Clear cell carcinoma, *MC* Mucinous carcinoma, *WT* Wild-type, *MT* Mutant

## Discussion

Somatic oncogenic mutations are actionable mutations in EOC patients. Validated mutations have been reported in 71–89% of EOC tumors using a cancer panel [14, 15], and the current study had a similar rate (82.5%). Sixty-six of the 80 EOC patients had one or more somatic mutations in either one or multiple genes (Table 1). We examined the association between somatic mutation frequencies and four histological subtypes of EOC. EC was found to have the highest frequency of mutations in the present study (Fig. 2). As a consequence of mismatch repair (MMR) deficiency due to mutations or methylation in MMR protein-encoding genes, tumors with MSI accumulate high numbers of mutations. EC tumors may have had higher MSI than other histological tumors.

We here examined the relationships between the five most frequently mutated genes and clinicopathological features or prognosis in EOC patients. p53 proteins involve in cell-cycle checkpoints, senescence, DNA repair, apoptosis and cellular stress responses. The *TP53* gene is one of the most commonly inactivated tumor suppressors in human cancer [16]. *TP53* was also identified to be mutated at the highest frequency in the current study (Fig. 1). In particular, *TP53* mutations were most frequently observed in SC (78%, 25/32) among the four histological subtypes (Table 2). According to The Cancer Genome Atlas (TCGA) Research Network, *TP53* mutations occur in 96% of HG-SC tumors [8]. The mutation spectrum completely separates HG-SC from other histological subtypes of EOC; in HG-SC, somatic mutations other than *TP53* mutations are rare. While it is well accepted that *TP53* mutation is an essential event in the genesis of HG-SC, we showed that mutant p53 was often seen in MC (57.1%, 4/7), which is consistent with previous studies [9, 17]. On the other hands, the frequencies of *TP53* mutation in CCC 9.5% (2/21) and EC 20% (4/20) were lower. Cybulska P and Kim SI reported that the frequency of *TP53* mutations was 17% (6/36) and 13% (2/15) in EC and CCC, respectively [10, 11]. The association of *TP53* mutations with late-stage EOC was statistically significant in the current study. The reason is that most of HG-SC with *TP53* mutations are diagnosed in an advanced stage, and the other histological subtypes (EC, CCC, and MC) that more commonly present at an early stage have a much lower incidence of *TP53* mutations. *TP53* mutations had marginally significant associations with poor PFS in the present study. Furthermore, patients with mutations only in *TP53* had a significantly worse PFS compared to those with no *TP53* mutations and those with *TP53* mutations with other mutations (Fig. 3d). Although *TP53* mutation was often seen in MC (57.1%, 4/7), all patients with MC harbored *KRAS* mutation. Table 3 shows that patients with HG-SC had significantly worse PFS than patients with other histological subtypes. Since patients with mutation only in *TP53* had a high rate of HG-SC (Fig. 1), they are predicted to have a poor prognosis.

PIK3CA encodes a lipid kinase involved in multiple signaling pathways that influence cellular functions such as growth, death, and proliferation. *PIK3CA* mutations occur in about 13% of solid tumors [18], and in our study, they occurred in 27.5% (22/80) of the EOC, which was similar to a recent study [19]. As for histological subtypes of EOC, *PIK3CA* mutation is rare in HG-SC and MC, although may be seen in up to 40% of CCC and EC [20], similar to our results (Table 2). *PIK3CA* mutations had significant associations with favorable PFS (*P* = 0.047) (Fig. 3a). In particular, we found *PIK3CA* mutations had more significant associations with favorable PFS than *PIK3CA* wild-type in the in the EC subtype (*P* = 0.012) (Fig. 3b). This significant prognostic value of *PIK3CA* mutation status in ECs is potentially interesting, and, to the best of our knowledge, has not been reported. In previous reports, PI3K pathway activation including the *PIK3CA* mutation was associated with favorable prognosis in CCC [21, 22]. Although *PIK3CA* mutation status was not a significant prognostic factor in CCC in our study, further studies with larger study populations are necessary to evaluate whether inactivation of PIK3CA is a useful biomarker.

The *KRAS* gene belongs to the Ras family of oncogenes and encodes the K-Ras protein, which is a part of the tyrosine kinase signaling RAS/MAPK pathway. *KRAS* mutations are the most frequent mutations in human cancer, and are found in up to 25% of all human cancers [23]. In the results of our study, *KRAS* mutations were detected in 23.8% (19/80) and correlated with early-stage, MC and better PFS. In the previous studies*, KRAS* mutations were reported to be related to type I histology types, especially their high frequency was reported in MCs and low-grade SCs [20, 24, 25]. In the present study, all of the MC tumors were observed to have *KRAS* mutations. As for prognosis, *KRAS* mutations have been proven to present in type I tumors characterized by early-stage and indolent behavior, and hence, generally associated with a more favorable survival [25].

PTEN inactivation results in abnormal cell growth and apoptosis escape. *PTEN* is known to be one of the most frequently mutated tumor suppressor genes in human cancer. Among several studies in EOC, the frequencies of *PTEN* mutations have been reported to be 5–21% [26–28], and said frequency in the present study was 10% (8/80). Regarding histological subtypes, *PTEN* mutations have been identified predominantly in type I, but not in type II [29]. In the current study, somatic mutations in *PTEN* were most frequently observed in EC (5/20, 25%). Dinulescu et al. reported that the combination of *PTEN* and *KRAS* mutations in the ovary induced invasive and widely metastatic EC [30]. They also suggest that aberrant activation of the PI3K/Akt/mTOR pathway may lead to the tumorigenesis and development of EC.

The β-catenin (CTNNB1) inactivation results in the activation of the Wnt signaling pathway, which plays an important role in the regulation of cell proliferation, differentiation tissue homeostasis, migration, embryonic development, cell fate determination, and self-renewal in stem cells [31]. Aberrant activation of Wnt signaling has been specifically shown to be associated with numerous malignancies [32]. Somatic mutations of *CTNNB1* were frequently observed in EC (30%, 6/20) and CCC (9.5%, 2/21) in the present study (Table 2). *CTNNB1* mutations are the most common genetic alterations identified in EC [11]. Not as much as EC tumors, *CTNNB1* mutations are present in approximately 10% of CCC tumors [33]. On the other hand, no *CTNNB1* mutations were observed in SC or MC in our study, which is similar to previous reports [8, 9], and this tendency was also observed in *PIK3CA* or *PTEN* mutations in our study. Lac et al. described hotspot mutations in *KRAS*, *ERBB2*, *PIK3CA* and *CTNNB1*, as well as heterogeneous *PTEN* loss and *ARID1A* loss in endometriosis [34]. Since it is thought that endometriosis-associated cancers include CCC and EC, gene mutations in endometriosis and these lesions may be similar [35, 36]. The associations of *CTNNB1* mutations with younger age and early-stage were significant in the present study. The reason is that the patients with CCC and EC, which are included in type I, were younger than those with HG-SC, which is included in type II. Interestingly, this tendency was also observed in uterine endometrial cancer [37].

The main limitations of our study were its retrospective design, the small sample size, histological heterogeneity, and short follow-up period. Therefore, a prospective study using a larger sample size with long-term follow-up would likely provide more reliable results. Another limitation of the current study was that our mutation analysis may have lacked any relevant targetable mutations due to a small hotspot panel. The use of genome-wide sequencing such as whole exome or genome sequencing would make it possible to detect more actionable gene mutations. Finally, we did not evaluate homologous recombination deficiency (HRD), *BRCA1/2* mutations and deficient MMR (dMMR). TCGA suggests that approximately 50% of HG-SC indicate HRD due to genetic and epigenetic alterations of HR pathway genes [8]. *BRCA1/2* mutations are the most common genetic cause of HRD. Approximately 15% of EOC cases are associated with germline *BRCA1/2* pathogenic variants, and additional 5% show somatic *BRCA1/2* mutations [38, 39]. Although a total of approximately 20% of *BRCA1/2* mutations should have been detected in the EOC tumors, the gene panel we used did not carry *BRCA1/2* genes. dMMR is estimated to occur in approximately 10% of EOC [40]. The reported frequency of MSI in EC ranges from 10 to 19% [11, 41, 42]. Rambau PF et al. have reported that dMMR was detected in 13.8% of EC and 2.4% of CCC, but not MC or SC using immunohistochemistry for MMR proteins [43]. Although we did not perform MSI assay or immunohistochemistry for MMR proteins in the present study, the result that EC had the highest frequency of mutations implied that EC tumors have the highest frequency of dMMR among histological subtypes of EOC [44]. Since HRD, including *BRCA1/2* mutations, and dMMR have recently emerged as predictive biomarkers for the choice of poly (ADP-ribose) polymerase inhibitor and immune checkpoint inhibitor, respectively, these analyses should have been performed in our study.

## Conclusion

We demonstrated that our comprehensive gene mutation profiles using targeted next generation sequencing panel in EOC are feasible. Although the clinical significance of somatic oncogenic mutations in EOC patients was similar to that detected in previous studies, we revealed the associations between *PIK3CA* mutation and better PFS in EC and between mutations only in *TP53* and worse PFS. This work will be useful to understand and evaluate the molecular features of EOC patients, and may help to establish future novel treatment strategies that improve outcome in EOC patients.

## Supplementary Information

**Additional file 1.** Summary of somatic oncogenic mutation status in 80 patients with epithelial ovarian cancer.

## Data Availability

The datasets used and analyzed during the present study are available from the corresponding author on reasonable request.
